# Effect of left atrial and ventricular abnormalities on renal transplant recipient outcome—a single-center study

**DOI:** 10.1186/s13737-014-0020-6

**Published:** 2014-12-03

**Authors:** Rajan K Patel, Christopher Pennington, Kathryn K Stevens, Alison Taylor, Keith Gillis, Elaine Rutherford, Nicola Johnston, Alan G Jardine, Patrick B Mark

**Affiliations:** BHF Glasgow Cardiovascular Research, University of Glasgow, 126 University Place, Glasgow, G12 8TA UK; Department of Renal Medicine, Western Infirmary, Dumbarton Road, Glasgow, G11 6NT UK; Department of Cardiology, Western Infirmary, Dumbarton Road, Glasgow, G11 6NT UK

**Keywords:** Outcome, Renal transplantation, Left atrial and ventricular function

## Abstract

**Background:**

Premature cardiovascular (CV) death is the commonest cause of death in renal transplant recipients. Abnormalities of left ventricular (LV) structure (collectively termed uremic cardiomyopathy) and left atrial (LA) dilation, a marker of fluid status and diastolic function, are risk factors for reduced survival in patients with end stage renal disease (ESRD). In the present analysis, we studied the impact of pre-transplant LA and LV abnormalities on survival after successful renal transplantation (RT).

**Methods:**

One hundred nineteen renal transplant recipients (first transplant, deceased donors) underwent cardiovascular MRI (CMR) as part of CV screening prior to inclusion on the waiting list. Data regarding transplant function and patient survival after transplantation were collected.

**Results:**

Median post-transplant follow-up was 4.3 years (interquartile range (IQR) 1.9, 6.2). During the post-transplant period, 13 patients returned to dialysis after graft failure and 23 patients died with a functioning graft. Survival analyses, censoring for patients returning to dialysis, showed that pre-transplant LV hypertrophy and elevated LA volume were significantly associated with reduced survival after transplantation. Multivariate Cox regression analyses demonstrated that longer waiting time, poorer transplant function, presence of LV hypertrophy and higher LA volume on screening CMR and female sex were independent predictors of death in patients with a functioning transplant.

**Conclusions:**

Presence of LVH and higher LA volume are significant, independent predictors of death in patients who are wait-listed and proceed with renal transplantation.

## Background

Premature cardiovascular (CV) death is the commonest cause of mortality in patients with end stage renal disease (ESRD) requiring renal replacement therapy [1]. Abnormalities of the left ventricular (LV) structure and function, including left ventricular hypertrophy (LVH), left ventricular systolic dysfunction (LVSD) and left ventricular dilation, are common in ESRD patients and independently confer a poorer prognosis [2,3]. These changes have been collectively termed “uremic cardiomyopathy”. Furthermore, left atrial (LA) dilation (corrected for body surface area (BSA) or height) is an independent predictor of mortality in the general population and in hypertensive and ESRD patients [4,5]. Impaired left ventricular diastolic relaxation and filling, mitral valve disease and fluid overload contribute to increased left atrial volume (LAV) [6], which can reliably and reproducibly be measured on echocardiography and CMR using the biplane area-length method [7,8]. We have previously demonstrated LA dilation, in the absence of significant mitral valve disease, to be an independent predictor of death in ESRD patients with LVH [5].

Renal transplantation (RT) is the optimal form of renal replacement therapy (RRT) and is associated with reduced CV morbidity and mortality compared to patients who remain on the transplant waiting list [9]. However, CV death is more common in RT recipients (RTRs) compared to the general population even in the presence of good transplant function [9-11]. Attempts to improve CV survival in RTRs have focused on modifying conventional CV risk factors, such as dyslipidemia, diabetes mellitus and hypertension after transplantation with varying degrees of success [12]. Pre-transplant factors have also been associated with adverse graft and recipient outcome including longer waiting list time, recipient age and greater co-morbidity [13,14]. Presence of uremic cardiomyopathy may be associated with adverse prognosis in RTR, and therapies aimed at reducing LV abnormalities prior to transplantation may help recipient and graft survival [3]. In addition, very few studies have investigated the effect of LA dilation on RTR survival [15].

Initial echocardiography studies demonstrated improved extracellular fluid control and regression of LVH which may account for improved CV outcome in ESRD patients after renal transplantation [16]. However, echocardiography overestimates LV mass in ESRD patients due to intravascular volume fluctuations, distortion of LV geometry and the reliance of standard estimates of LV mass on chamber diameters. Cardiovascular MRI (CMR) provides more detailed, volume independent, measurement of cardiac structure and is considered the most accurate technique for assessing ventricular dimensions in patients with ESRD. We have previously demonstrated no regression of LV abnormalities in RTRs up to 2.4 years post-transplantation using CMR [17]; the inference being that reduction in LV mass after transplantation reported by echocardiography is an artefact of normalisation of intravascular volume.

The aim of the current study was to assess the effect of pre-transplant LV and LA abnormalities measured by CMR on post-transplant outcomes.

## Methods

### Patients

Since 2002 [18,19], we have used CMR as part of the standard assessment of patients for renal transplantation. Patients were referred by transplant nephrologists or surgeons based on previously described criteria [19]. The renal transplant unit at the Western Infirmary, Glasgow, provides transplant services to a population of 2.8 million people in the west of Scotland. The transplant waiting list has 300–400 patients at any time point; approximately 100–120 new patients are wait-listed and approximately 95 deceased donor adult transplants are performed annually. CMR assessment was performed on 119 patients with CKD stage 5 receiving (hemo- or peritoneal) dialysis who subsequently underwent successful renal transplantation. Only patients receiving their first transplant and grafts from deceased (donation before cardiac death) donors were included in the study. This study was approved by the west of Scotland Research Ethics Committee (as part of previously published studies [5,19]), and all patients gave written, informed consent.

Patients who received a transplant before requiring dialysis were excluded. To ensure that only non-valvular causes of LA dilation were assessed, patients with mild to severe mitral valve disease on echocardiography, based on American Society of Echocardiography guidelines [20], were excluded from the study. No patients suffered a CV event between assessment and transplantation. Date and cause of patient death were detected from electronic patient records.

### CMR technique and analysis

Non-gadolinium contrast CMR was performed using a 1.5 Tesla MRI scanner (Sonata, Siemens, Erlangan, Germany) with LV mass and function assessed as previously described [21]. Scans were consistently performed 24 h after the end of the last dialysis session in hemodialysis patients. A fast imaging with steady-state precession (TrueFISP) sequence was used to acquire cine images in long axis planes (vertical long axis, horizontal long axis, left ventricular outflow tract) followed by sequential short axis LV cine loops (8-mm slice thickness, 2-mm gap between slices) from the atrioventicular ring to the apex. Imaging parameters, which were standardised for all subjects, included repetition time (TR)/echo time (TE)/flip angle/voxel size/field of view (FoV) = 3.14 ms/1.6 ms/60°/2.2 1.3 8.0 mm/340 mm. LV mass was analysed by two observers, blinded to patient clinical characteristics, from short axis cine loops using manual tracing of epicardial and endocardial end-systolic and end-diastolic contours. End-systolic and end-diastolic volumes and LV mass were calculated using analysis software (Argus, Siemens, Erlangen, Germany). LVH was defined as left ventricular mass index (LV mass/body surface area; LVMI) >84.1 g/m^2^ (male) or >66.8 g/m^2^ (female), and LVSD was defined as LV ejection fraction (LVEF) <55%. LV dilation was defined as end diastolic volume/body surface area (EDV/BSA) >111.7 ml/m^2^ (male) or 99.3 ml/m^2^ (female) or end systolic volume/body surface area (ESV/BSA) >92.8 ml/m^2^ (male) or 70.3 ml/m^2^ (female) [22].

The biplane area-length method for ellipsoid bodies was used [7] to measure LAV. Horizontal and vertical long axis cine images were used to obtain images of the left atrium at maximal filling. The atrial lengths and areas were measured from both views, and LAV was calculated. LAV was corrected for body surface area (LAV/BSA). Patients were categorised into two LAV groups according to mean LAV (i.e. low = less than mean LAV and high = greater than or equal to mean LAV). Left atrial appendages were included in these measurements.

### Mitral valve inflow Doppler velocity measurement

Echocardiography was performed by an experienced echocardiographer using an Acuson Sequoia C512 machine (Siemens Medical, Mountainview, CA, USA). Diastolic function was assessed using pulsed wave Doppler [23,24] from apical four chamber views to measure the ratio of early (E) to late (A) mitral inflow peak flow velocity (E/A ratio).

### Clinical and blood result data collection

Demographic information and clinical history were recorded at the time of CMR. Electronic patient record review was performed to retrieve post-transplant information including medication and outpatient clinic systolic and diastolic blood pressure (SBP and DBP, respectively) 60 days prior to the date of censor or patient death. Biochemical and hematological blood results were collected at the time of CMR. In addition, data regarding transplant function (as assessed by serum creatinine) were collected for the 60 days up to the date of death or censor (22nd March 2011) accordingly. Data regarding patients initially listed but subsequently suspended were not available for analyses. Delayed graft function was defined as requirement for hemodialysis within 7 days of transplantation. Indication biopsy findings were used to determine presence of biopsy-proven acute rejection (BPAR) according to standard Banff classification.

### Statistical analyses

Data are described as mean and standard deviation (for normally distributed data) or median (interquartile range, IQR) for non-normal data. Survival analyses were performed from the time of transplantation and censored for graft failure and return to dialysis. Comparisons of mean survival (±standard deviation) for different cardiac parameters are shown as Kaplan-Meier graphs (with statistical comparison using the log rank test). These data were also analysed by Cox multivariate survival analysis to assess the influence of multiple clinical and cardiac variables on outcome. Variables identified as significantly influential on outcome by univariate analysis were entered into a backward stepwise regression model. All analyses were performed using SPSS v19.0 (SPSS Inc, www.spss.com). Log minus log plots of survival were performed to check proportionality assumptions of the Cox model.

## Results

Using data from 402 ESRD patients assessed for renal transplantation between 2002 and 2010, 326 patients were listed for transplantation and 119 underwent renal transplantation. As stated before, these patients were referred for assessment for first donation before cardiac death renal transplant, having been deemed “moderate” CV risk based on specific criteria [19] by the referring transplant physician or surgeon.

### Cardiac assessment parameters

Table 1 shows patient data gathered at the time of cardiac assessment including mode of RRT, past medical history and cardiac drug history. The mean age at assessment was 50.5 (±10.2) years and the majority of patients assessed were male (69.7%). Median time from starting dialysis to cardiac assessment was 2.2 years (IQR 0.6, 5.2). There was high prevalence of patients with diabetes (61.3%) and past history of ischemic heart disease (14.3%) who underwent cardiac assessment. As we have demonstrated before, LVH was common (65.5%) on CMR assessment of these patients. CMR data are also shown in Table 1. Mean LAV/BSA was 32.1 (±7.7) ml/m^2^.

**Table 1:** **Demographic, clinical and drug data for patients at pre-transplant cardiac assessment**

**Variable**	**Total ** ***N*** ** = 119**	**Alive** ***N*** ** = 96**	**Dead** ***N*** ** = 23**	***p***
Age at CMR (years)	50.5 (±10.2)	49.8 (±9.7)	53.7 (±11.5)	0.05*
Male (%)	83 (69.7)	71 (74.0)	12 (52.2)	0.04*
BSA (m^2^)	1.84 (±0.3)	1.86 (±0.3)	1.77 (±0.2)	0.12
BMI (kg/m^2^)	25.0 (±4.7)	24.9 (±0.7)	25.4 (±4.5)	0.12
Systolic BP (mmHg)	138 (±23)	137 (±23)	141 (±24.2)	0.38
Diastolic BP (mmHg)	82 (±12.7)	82 (±13)	81 (±13)	0.77
RRT time to CMR (years)	2.2 (0.6, 5.2)	1.7 (0.7, 3.9)	2.5 (0.6, 5.8)	0.57
RRT				
HD	100 (84.0)	84 (87.5)	16 (69.5)	0.91
PD	19 (16.0)	11 (11.4)	8 (34.7)	
Diabetes mellitus	73 (61.3)	61 (63.5)	12 (52.2)	0.32
Ischemic heart disease	17 (14.3)	13 (13.5)	4 (17.4)	0.64
Heart failure	6 (5.0)	5 (5.2)	1 (4.3)	0.86
Cerebrovascular disease	12 (10.1)	6 (6.3)	6 (26.1)	0.005*
Peripheral vascular disease	7 (5.9)	4 (4.2)	3 (13.0)	0.10
Dyslipidemia	34 (28.6)	26 (27.1)	8 (34.8)	0.46
Smoking				
Never	52 (43.7)	44 (45.8)	8 (34.8)	0.14
Current/Ex smoker	67 (56.3)	52 (54.2)	15 (65.2)	
Epo receptor agonist	92 (77.3)	74 (77.9)	18 (78.3)	0.97
β adrenoceptor blocker	40 (33.6)	35 (37.6)	5 (21.7)	0.15
Aspirin	32 (26.9)	26 (28.0)	6 (26.1)	0.86
Warfarin	5 (4.2)	2 (2.2)	3 (13.0)	0.08
ACEI/ARB pre-transplant	25 (21.0)	22 (23.4)	3 (13.0)	0.28
Diuretic	30 (25.2)	24 (25.8)	6 (26.1)	0.98
Calcium channel blocker	29 (24.4)	23 (24.7)	6 (26.1)	0.89
α adrenoceptor blocker	10 (8.4)	9 (9.7)	1 (4.3)	0.42
Statin	34 (28.6)	26 (28.0)	8 (34.8)	0.52
Hemoglobin (g/l)	117 (±17)	117 (±1.7)	117 (±1.8)	0.97
CRP	6.0 (4.0, 41.2)	6.0 (4.0, 12.0)	19 (4.0, 49.5)	0.19
Corrected calcium (mmol/l)	2.42 (±0.4)	2.41 (±0.4)	2.42 (±0.5)	0.97
Phosphate (mmol/l)	1.67 (±0.4)	1.71 (±0.4)	1.52 (±0.5)	0.30
Albumin (g/l)	41.5 (±4.3)	42.5 (±3.7)	37.5 (±4.7)	0.008*
Ejection fraction (%)	66.3 (±10.2)	66.3 (±9.5)	66.4 (±12.3)	0.99
LVSD on MRI (EF < 55%)	16 (13.4)	11 (11.5)	5 (21.7)	0.19
Myocardial mass/BSA (g/m^2^)	96.4 (±31.7)	95.2 (±31.7)	101.6 (±32.4)	0.38
LVH	78 (65.5)	58 (60.4)	20 (87.0)	0.16
ESV/BSA (ml/m^2^)	25.1 (±14.8)	72.4 (±25.7)	73.5 (±25.2)	0.85
EDV/BSA (ml/m^2^)	72.7 (±25.5)	24.9 (±13.8)	25.9 (±18.9)	0.77
LV dilation	14 (13.4)	11 (11.5)	3 (13.0)	0.83
LA volume/BSA (ml/m^2^)	32.1 (±7.7)	31.0 (±6.7)	36.4 (±9.1)	0.01*

Data are numbers with percentage in parentheses, mean ± standard deviation or median (interquartile range). Tests of significance are *t*-test (parametric), Mann-Whitney (non-parametric) and Chi-square. Abbreviations: *CMR* cardiovascular MRI, *RRT* renal replacement therapy, *HD* hemodialysis, *PD* peritoneal dialysis, *Epo* erythropoietin stimulating agent, *ACEI/ARB* angiotensin-converting enzyme inhibitor/angiotensin II receptor blocker, *BSA* body surface area, *BMI* body mass index. *p < 0.05.

### Peri- and post-transplant parameters

The median time from cardiac assessment to transplantation was 2.6 years (IQR 1.1, 4.3 years) and time on transplant waiting list was 2.4 (IQR 1.1, 4.2 years). Thirteen (10.9%) patients developed graft failure and returned to dialysis. The median transplant follow-up, censored for return to dialysis, was 4.3 years (IQR 1.9, 6.2 years). Table 2 shows immunosuppressive therapy for patients at the time of censoring or death. Mean creatinine from 60 days prior to death or censoring was 188 (±38) μmol/l. Forty two (35.3%) RTRs developed delayed graft function (DGF) and sixteen (13.4%) developed biopsy-proven acute rejection during follow-up.

**Table 2:** **Peri- and post-transplantation data**

**Variable**	**Total** ***N*** ** = 119**	**Alive** ***N*** **= 96**	**Dead** ***N*** **= 23**	***p***
Age at transplantation (years)	53.1 (±10.5)	52.7 (±10.0)	55.0 (±12.2)	0.33
Assessment to transplant (years)	2.6 (1.1, 4.3)	1.3 (0.8, 3.5)	2.6 (1.2, 4.3)	0.09
Waiting list time (years)	2.4 (1.1, 4.2)	1.8 (0.7, 3.5)	2.5 (1.1, 4.3)	0.02*
Mycophenolic acid	62 (52.1)	53 (55.2)	9 (39.1)	0.14
Azathioprine	45 (37.9)	31 (32.2)	14 (60.8)	0.07
Prednisolone	119	96 (100)	23 (100)	
Tacrolimus	57 (47.9)	46 (47.9)	11 (47.8)	0.92
Cyclosporin	45 (37.9)	34 (35.4)	11 (47.8)	0.64
Sirolimus	3 (2.5)	3 (3.1)	0 (0)	
ACEI/ARB therapy post-transplant	22 (17.6)	20 (20.8)	2 (8.7)	0.18
Mean creatinine at censor (μmol/l)	188 (±38.2)	173 (±116)	253 (±151)	0.02
Biopsy-proven acute rejection	16 (13.4)	15 (15.6)	1 (4.3)	0.15
Delayed graft function	42 (35.3)	29 (30.2)	13 (56.5)	0.02*

Data are numbers with percentage in parentheses, mean ± standard deviation or median (interquartile range). Tests of significance are *t*-test (parametric), Mann-Whitney (non-parametric) and Chi-square. *ACEI/ARB* angiotensin-converting enzyme inhibitor/angiotensin II receptor blocker. *p < 0.05.

### Outcome after transplantation

We initially examined the effect of patient characteristics on outcome in RTRs (Table 1). Twenty three RTRs died during the post-transplant follow-up period. Cause of death was cardiac in 9 (39.1%), sepsis in 7 (30.4%), malignancy in 2 (8.7%) and unknown in 3 (13.0%) cases. “Other”, non-defined, causes accounted for 2 (8.7%) deaths. There were significant associations between patient death and older age, female sex, past history of cerebrovascular disease and lower serum albumin at the time of cardiac assessment. LAV/BSA was significantly higher in patients who died during follow-up (*p* = 0.01). In addition, longer duration on the deceased donor renal transplant waiting list and poor transplant function prior (60 days) to censor or death were significantly associated with death after transplantation (Table 2). There was no significant difference between the rate of angiotensin-converting enzyme inhibitor (ACEI) or angiotensin II receptor blocker (ARB) usage (pre- and post-transplant) between survivors and the patients who died during follow-up.

**Figure 1:**
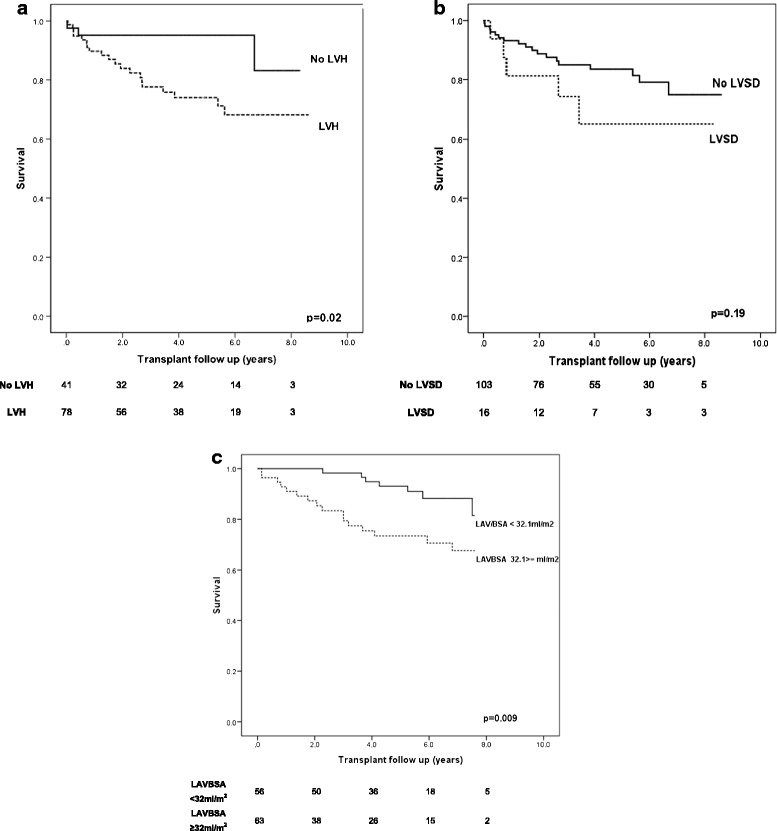
**Kaplan Meier survival curve (censored for graft failure) according to the presence of (1a) left ventricular hypertrophy (LVH), (1b) left ventricular systolic dysfunction and (1c) left atrial dilation.**

We investigated the effect of left ventricular and atrial abnormalities detected by CMR on RTR outcome censored for return to dialysis (Figure 1a–c). The presence of LVH on CMR at cardiac assessment was significantly associated with a reduction in mean survival time (Figure 1a- no LVH 7.7 ± 2.0 years vs. LVH 6.6 ± 3.3 years; *p* = 0.023). The presence of LVSD or LV dilation was not significantly associated with reduction in mean survival time (Figure 1b- No LVSD 7.2 ± 2.9 years vs. LVSD 6.0 ± 3.4 years; *p* = 0.19. No LV dilation 7.1 ± 3.1 years vs. LV dilation 6.1 ± 2.7 years; *p* = 0.80). Patients were categorised into two groups according to mean LAV/BSA (less than or greater than or equal to 32.1 ml/m^2^). Higher LAV/BSA was significantly associated with reduced survival time (Figure 1c- < 32.1 ml/m^2^ 7.2 ± 1.2 years vs. ≥32.1 ml/m^2^ 6.1 ± 2.6 years; *p* = 0.009). Compared to patients with normal cardiac stucture or LA dilation alone, there was a trend towards poorer survival in patients with LA dilation and LVH or LVSD, but this did not reach statistical significance (data not shown).

### Survival analyses

Table 3 shows univariate and multivariate Cox survival analyses for patient clinical and cardiac characteristics at the time of cardiac assessment. Univariate analyses showed that longer time on the deceased donor renal transplant waiting list, poorer transplant function (60 days pre censor), presence of LVH, higher LAV/BSA, female sex, past history of cerebral and peripheral vascular disease, older age at transplantation and development of delayed graft function were significantly associated with death. Multivariate analysis (Table 3) demonstrated that longer time on the renal transplant waiting list, poorer transplant function, presence of LVH and higher LAV/BSA, female sex and delayed graft function were independently associated with death after renal transplantation.

**Table 3:** **Results of univariate and multivariate Cox regression survival analyses of all RTRs; all-cause mortality (** ***N*** **= 23) is the dependable variable**

**Variable**	**Univariate analyses**			**Multivariate analyses**		
	**HR**	**95% CI**	***p***	**HR**	**95% CI**	***p***
Time on waiting list (per year)	1.27	1.00-1.62	0.05*	1.62	1.07–1.78	0.02*
Creatinine (per 10 μmol/l)	1.03	1.01–1.06	0.01*	1.07	1.03–1.11	<0.001*
LVH	3.78	1.12–12.72	0.03*	6.23	1.18–22.31	0.004*
Male	0.43	0.19–0.98	0.04*	0.23	0.11–0.65	0.02*
LAV/BSA above 32.1 ml/m^2^	3.11	1.27–7.57	0.01*	6.60	1.70–18.58	0.02*
Delayed graft function	2.42	1.08–5.64	0.03	3.12	1.02–9.57	0.05*
Cerebrovascular disease	6.76	2.58–17.78	0.05*	3.12	0.91–23.7	0.07
Peripheral vascular disease	3.36	1.00–11.37	0.05*	7.38	0.51–52.3	0.21
Age at transplantation (per year)	1.04	1.00–1.08	0.05*	1.02	0.96–1.26	0.43
BMI (per kg/m^2^)	1.02	0.94–1.12	0.69			
Biopsy-proven acute rejection	2.76	0.37–5.68	0.21			
LVSD	2.38	0.88–6.47	0.08			
Ischemic heart disease	1.33	0.45–3.91	0.61			
LV dilation	1.16	0.34–3.90	0.82			
Diabetes mellitus	0.59	0.25–1.31	0.19			
Heart failure	0.86	0.12–6.40	0.88			
Current/Ex smoker (ref never)	1.58	0.67–3.72	0.30			
Systolic BP (per mmHg)	1.02	0.99–1.04	0.15			
Diastolic BP (per mmHg)	0.98	0.94–1.02	0.26			
Dyslipidemia	1.67	0.71–3.99	0.24			
RRT time to CMR (years)	0.92	0.81–1.05	0.21			
Previous RRT type						
PD	1.00					
HD	1.29	0.17–9.79	0.80			
Hemoglobin (per g/dl)	0.65	0.97–1.13	0.13			
Albumin (per g/l)	0.83	0.65–1.05	0.13			
Phosphate (per mmol/l)	0.43	0.07–2.75	0.37			
Calcium (per mmol/l)	5.45	0.11–12.54	0.40			
ACEI/ARB therapy pre-transplant	0.52	0.16–1.76	0.29			
ACEI/ARB therapy post-transplant	0.42	0.10–1.80	0.24			

Hazard ratios (95% confidence intervals (CI)) are shown. Variables highlighted with (*) were added to the multivariate Cox model. Abbreviations: *LV* left ventricle, *LVH* left ventricular hypertrophy, *LVSD* left ventricular systolic dysfunction, *BMI* body mass index, *BP* blood pressure, *RRT* renal replacement therapy, *PD* peritoneal dialysis, *HD* hemodialysis. *p < 0.05

## Discussion

Renal transplantation is associated with a significant reduction of CV disease in patients with ESRD compared to patients who remain on the renal transplant waiting list [10]. Nonetheless, RTRs have three- to fivefold increased risk of premature CV morbidity compared to the general population, and CV disease is the leading cause of death and graft loss [25,26]. The pathogenesis of CVD in RTRs differs from the general population in that sudden, presumed arrhythmic, cardiac death and the sequelae of coronary artery disease have similar prevalence. The Assessment of LEscol in Renal Transplantation (ALERT) study demonstrated that, in RTRs, myocardial infarction was dependent upon lipids and conventional risk factors for CAD, whereas the risk factors for sudden cardiac death included renal dysfunction, blood pressure and LVH [12,27]. In an attempt to prolong post-transplant and graft survival, several studies have identified potential risk factors for CV disease amenable to modification. Most of these studies have focused on post-transplant factors, and few have determined whether modification of risk factors whilst on the transplant waiting list can have significant effect on recipient or graft outcome.

Against this background, we sought to determine factors associated with death after renal transplantation in a cohort of patients who underwent comprehensive CV assessment prior to their inclusion on the deceased donor renal transplant waiting list. To minimise confounding factors, only patients receiving their first transplant from deceased donors (death before cardiac death) were included in the analyses. In particular, we investigated the effect of CMR-measured left atrial and ventricular abnormalities on post-transplant survival. These abnormalities have previously been shown to be independent predictors of death in dialysis patients. Although these appear to improve after transplantation when measured by echocardiography [2,16], there are no significant reductions in LV mass, function or chamber size assessed by CMR which provides a volume-independent measurement of LV parameters [17]. These observations highlight the challenges of echocardiography and the effect of intravascular (and hence intraventricular) volume status when assessing LV parameters of ESRD patients including transplant recipients [28].

### Left ventricular abnormalities

Our data demonstrate that the presence of LVH at cardiac assessment was an independent predictor of death after renal transplantation. In addition, the presence of LVH was significantly associated with poorer survival (Figure 1a). Thus, LVH may be a potential therapeutic target to improve survival after transplantation. Unfortunately, interventions shown to regress LVH in patients with normal renal function are less successful in ESRD subjects. LVH after transplantation is associated with pre- and post-transplant hypertension (which is common and severe in RTRs even when graft function is good), side effects of immunosuppressive agents, extracellular fluid overload and vascular stiffness [29]. These features increase LV preload and afterload to varying degrees resulting in alteration in chamber stress (pressure and volume) and stimulation of LV wall architectural changes. In this study, left ventricular systolic dysfunction and dilation were not significantly associated with death or reduced survival time (Table 3 and Figure 1b, c), and this is most likely due to a small number of candidates with these abnormalities who subsequently received a renal transplant. Moreover, in a previous study of cardiac screening, we found that the presence of severe cardiac abnomalities often resulted in patients not being listed for transplantation [19].

Attempts to reduce LV mass and improve CV outcome after transplantation have aimed to control blood pressure. In a small randomised control trial investigating the effect of ACEI on long-term outcome of RTRs, treatment was significantly associated with better CV and general outcome with no significant adverse effects to transplant function [30]. In our study, treatment with ARB/ACEI before or after transplantation was not significantly associated with RTR survival (Tables 1, 2 and 3). Furthermore, modification of immunosuppressive regimen (including early withdrawal of glucocorticoid therapy, minimisation of calcineurin antagonists) has been successful with significant reduction in blood pressure [31,32], although it is unclear whether this will have an effect on CV survival. More recently, the use of sirolimus, in place of CNIs, was associated with regression of LVH [33,34] in a small non-randomised trial in RTRs. In addition, initial results from the BENEFIT trial (belatacept versus high-dose cyclosporin), looking at blood pressure, lipids and NODAT showed that patients on belatacept had an improved metabolic risk profile at 12 months [35]. In general, transplant clinicians and transplant recipients may be reluctant to change immunosuppressive regimen or implement specific antihypertensive therapy to achieve blood pressure control, because of the perceived immunological and vascular risk and possibility of jeopardising graft function. Newer strategies to control blood pressure in RTRs may allow sustained blood pressure control, improve LV parameters and reduce post transplant mortality without the need for a significant change to medications.

### Left atrial dilation

We have previously shown that LA dilation measured by CMR confers poorer prognosis in hemodialysis patients with LVH and no significant mitral valve disease. Elevated LA volumes are a consequence of chronic elevated ventricular filling pressures, impaired LV relaxation during diastole (diastolic dysfunction) and volume overload in dialysis patients. However, in RTRs, fluid overload is avoided and improved due to good graft function and physiological response to euvolemia.

In this study, LA dilation before transplantation was significantly associated with reduced post-transplant survival. Furthermore, elevated LAV was associated with death independently from LVH. Taken together, these data suggest LA changes (associated with chronic abnormalities of ventricular and atrial pressure) and its adverse effect on prognosis may persist after transplantation despite improvement in fluid status. Moreover, LA dilation is identified as a potentially remediable risk factor, perhaps by the use of strategies that improve LV compliance and associated diastolic dysfunction.

In both living and deceased donor renal transplantation, time spent on the transplant waiting list is independently associated with adverse outcome after transplantation [13]. Unfortunately, this is unlikely to change as attempts to reduce waiting list times for deceased donor transplantation have been unsuccessful due to the discrepancy between kidney transplant demand and organ supply. However, strategies to reduce waiting time by, for example, living donation and pre-emptive transplantation are likely to have long-term benefits. Poorer graft function, measured by mean serum creatinine in the preceding 60 days prior to censor, was independently associated with post-transplant death. However, it is difficult to determine from these data whether this was a significant causative factor to death or a marker of worsening morbidity. Finally, female sex was an independent predictor of adverse outcome in this study, and this is most likely due to the low number of women in our cohort who subsequently underwent transplantation. Compared to other studies, the rates of graft loss and patient death were higher, most likely reflecting higher degree of co-morbidity in patients referred for CV assessment.

This study has limitations. The time from CV assessment to transplantation was not consistent and may act as a confounder for prognostic cardiovascular modelling. In addition, there is a lack of post-transplant LV and LA data. However, our previous study demonstrating no changes in CMR measured LV dimensions after transplantation suggests that abnormalities present at assessment prior to transplantation persist during the follow-up of RTRs [9].

The aim of this study was to highlight potentially modifiable CV risk factors before and after transplantation. Newer strategies are required to improve uremic cardiomyopathy and LA abnormalities in patients with advanced stages of chronic kidney disease, given its effect on patient outcome even after renal transplantation. However, the presence of LVH or LA dilation at the time of CV assessment should not act as a barrier to transplant listing given that renal transplantation is one of the few interventions reliably shown to improve outcomes in patients with CKD 5 [9].

## Conclusions

In conclusion, the presence of LVH and LA dilation are significant predictors of death in patients who are listed and subsequently proceed with renal transplantation. Future studies should target LA dilation and LVH prevention and progression whilst awaiting renal transplantation in an attempt to reduce CV mortality in the long-term follow-up of these patients.

## Abbreviations

CV: Cardiovascular
LV: Left ventricle
LA: Left atrial
CMR: Cardiovascular MRI
ESRD: End stage renal disease
LVSD: Left ventricular systolic dysfunction
LVH: Left ventricular hypertrophy
BSA: Body surface area
RRT: Renal replacement therapy
RTR: Renal transplant recipients

## References

[1] Baigent C, Burbury K, Wheeler D (2000). Premature cardiovascular disease in chronic renal failure. Lancet.

[2] Parfrey PS, Foley RN, Harnett JD, Kent GM, Murray DC, Barre PE (1996). Outcome and risk factors for left ventricular disorders in chronic uraemia. Nephrol Dial Transplant.

[3] McGregor E, Jardine AG, Murray LS, Dargie HJ, Rodger RS, Junor BJ, McMillan MA, Briggs JD (1998). Pre-operative echocardiographic abnormalities and adverse outcome following renal transplantation. Nephrol Dial Transplant.

[4] Tripepi G, Benedetto FA, Mallamaci F, Tripepi R, Malatino L, Zoccali C (2007). Left atrial volume monitoring and cardiovascular risk in patients with end-stage renal disease: a prospective cohort study. J Am Soc Nephrol.

[5] Patel RK, Jardine AG, Mark PB, Cunningham AF, Steedman T, Powell JR, McQuarrie EP, Stevens KK, Dargie HJ, Jardine AG (2010). Association of left atrial volume with mortality among ESRD patients with left ventricular hypertrophy referred for kidney transplantation. Am J Kidney Dis.

[6] Abhayaratna WP, Seward JB, Appleton CP, Douglas PS, Oh JK, Tajik AJ, Tsang TS (2006). Left atrial size: physiologic determinants and clinical applications. J Am Coll Cardiol.

[7] Lang RM, Bierig M, Devereux RB, Flachskampf FA, Foster E, Pellikka PA, Picard MH, Roman MJ, Seward J, Shanewise J, Solomon S, Spencer KT, St John Sutton M, Stewart W (2006). Recommendations for chamber quantification. Eur J Echocardiogr.

[8] Sievers B, Kirchberg S, Addo M, Bakan A, Brandts B, Trappe HJ (2004). Assessment of left atrial volumes in sinus rhythm and atrial fibrillation using the biplane area-length method and cardiovascular magnetic resonance imaging with TrueFISP. J Cardiovasc Magn Reson.

[9] Meier-Kriesche HU, Schold JD, Srinivas TR, Reed A, Kaplan B (2004). Kidney transplantation halts cardiovascular disease progression in patients with end-stage renal disease. Am J Transplant.

[10] Wolfe RA, Ashby VB, Milford EL, Ojo AO, Ettenger RE, Agodoa LY, Held PJ, Port FK (1999). Comparison of mortality in all patients on dialysis, patients on dialysis awaiting transplantation, and recipients of a first cadaveric transplant. N Engl J Med.

[11] Kasiske BL (1988). Risk factors for accelerated atherosclerosis in renal transplant recipients. Am J Med.

[12] Holdaas H, Fellström B, Cole E, Nyberg G, Olsson AG, Pedersen TR, Madsen S, Grönhagen-Riska C, Neumayer HH, Maes B, Ambühl P, Hartmann A, Staffler B, Jardine AG (2005). Long-term cardiac outcomes in renal transplant recipients receiving fluvastatin: the ALERT extension study. Am J Transplant.

[13] Meier-Kriesche HU, Kaplan B (2002). Waiting time on dialysis as the strongest modifiable risk factor for renal transplant outcomes: a paired donor kidney analysis. Transplantation.

[14] Arend SM, Mallat MJ, Westendorp RJ, van der Woude FJ, van Es LA (1997). Patient survival after renal transplantation; more than 25 years follow-up. Nephrol Dial Transplant.

[15] Kainz A, Goliasch G, Wiesbauer F, Binder T, Maurer G, Nesser HJ, Mascherbauer R, Ebner C, Kramar R, Wilflingseder J, Oberbauer R (2013). Left atrial diameter and survival among renal allograft recipients. Clin J Am Soc Nephrol.

[16] Montanaro D, Gropuzzo M, Tulissi P, Vallone C, Boscutti G, Mioni R, Risaliti A, Baccarani U, Adani GL, Sainz M, Lorenzin D, Bresadola F, Mioni G (2005). Effects of successful renal transplantation on left ventricular mass. Transplant Proc.

[17] Patel RK, Mark PB, Johnston N, McGregor E, Dargie HJ, Jardine AG (2008). Renal transplantation is not associated with regression of left ventricular hypertrophy: a magnetic resonance study. Clin J Am Soc Nephrol.

[18] Mark PB, Johnston N, Groenning BA, Foster JE, Blyth KG, Martin TN, Steedman T, Dargie HJ, Jardine AG (2006). Redefinition of uremic cardiomyopathy by contrast-enhanced cardiac magnetic resonance imaging. Kidney Int.

[19] Patel RK, Mark PB, Johnston N, McGeoch R, Lindsay M, Kingsmore DB, Dargie HJ, Jardine AG (2008). Prognostic value of cardiovascular screening in potential renal transplant recipients: a single-center prospective observational study. Am J Transplant.

[20] Bonow RO, Carabello BA, Kanu C, de Leon AC, Faxon DP, Freed MD, Gaasch WH, Lytle BW, Nishimura RA, O'Gara PT, O'Rourke RA, Otto CM, Shah PM, Shanewise JS, Smith SC, Jacobs AK, Adams CD, Anderson JL, Antman EM, Faxon DP, Fuster V, Halperin JL, Hiratzka LF, Hunt SA, Lytle BW, Nishimura R, Page RL, Riegel B (2006). ACC/AHA 2006 guidelines for the management of patients with valvular heart disease: a report of the American College of Cardiology/American Heart Association Task Force on Practice Guidelines (writing committee to revise the 1998 Guidelines for the Management of Patients With Valvular Heart Disease): developed in collaboration with the Society of Cardiovascular Anesthesiologists: endorsed by the Society for Cardiovascular Angiography and Interventions and the Society of Thoracic Surgeons. Circulation.

[21] Stewart GA, Mark PB, Johnston N, Foster JE, Cowan M, Rodger RS, Dargie HJ, Jardine AG (2004). Determinants of hypertension and left ventricular function in end stage renal failure: a pilot study using cardiovascular magnetic resonance imaging. Clin Physiol Funct Imaging.

[22] Alfakih K, Plein S, Thiele H, Jones T, Ridgway JP, Sivananthan MU (2003). Normal human left and right ventricular dimensions for MRI as assessed by turbo gradient echo and steady-state free precession imaging sequences. J Magn Reson Imaging.

[23] Appleton CP, Jensen JL, Hatle LK, Oh JK (1997). Doppler evaluation of left and right ventricular diastolic function: a technical guide for obtaining optimal flow velocity recordings. J Am Soc Echocardiogr.

[24] Nagueh SF, Appleton CP, Gillebert TC, Marino PN, Oh JK, Smiseth OA, Waggoner AD, Flachskampf FA, Pellikka PA, Evangelisa A (2009). Recommendations for the evaluation of left ventricular diastolic function by echocardiography. Eur J Echocardiogr.

[25] Kasiske BL, Malik MA, Herzog CA (2005). Risk-stratified screening for ischemic heart disease in kidney transplant candidates. Transplantation.

[26] Kidney Disease: Improving Global Outcomes (2009). KDIGO clinical practice guideline for the care of kidney transplant recipients. Am J Transplant.

[27] Fellström B, Holdaas H, Jardine AG, Holme I, Nyberg G, Fauchald P, Grönhagen-Riska C, Madsen S, Neumayer HH, Cole E, Maes B, Ambühl P, Olsson AG, Hartmann A, Logan JO, Pedersen TR (2004). Effect of Fluvastatin on renal end points in the assessment of lescol in renal transplant (ALERT) trial. Kidney Int.

[28] Stewart GA, Foster J, Cowan M, Rooney E, McDonagh T, Dargie HJ, Rodger RS, Jardine AG (1999). Echocardiography overestimates left ventricular mass in hemodialysis patients relative to magnetic resonance imaging. Kidney Int.

[29] Patel RK, Oliver S, Mark PB, Powell JR, McQuarrie EP, Traynor JP, Dargie HJ, Jardine AG (2009). Determinants of left ventricular mass and hypertrophy in hemodialysis patients assessed by cardiac magnetic resonance imaging. Clin J Am Soc Nephrol.

[30] Paoletti E, Bellino D, Marsano L, Cassottana P, Rolla D, Ratto E (2013). Effects of ACE inhibitors on long-term outcome of renal transplant recipients: a randomized controlled trial. Transplantation.

[31] Jardine AG, Gaston RS, Fellstrom BC, Holdaas H (2011). Prevention of cardiovascular disease in adult recipients of kidney transplants. Lancet.

[32] Marcen R (2009). Immunosuppressive drugs in kidney transplantation: impact on patient survival, and incidence of cardiovascular disease, malignancy and infection. Drugs.

[33] Paoletti E, Amidone M, Cassottana P, Gherzi M, Marsano L, Cannella G (2008). Effect of sirolimus on left ventricular hypertrophy in kidney transplant recipients: a 1-year nonrandomized controlled trial. Am J Kidney Dis.

[34] Paoletti E, Cannella G (2010). Regression of left ventricular hypertrophy in kidney transplant recipients: the potential role for inhibition of mammalian target of rapamycin. Transplant Proc.

[35] Vincenti F, Larsen CP, Alberu J, Bresnahan B, Garcia VD, Kothari J, Lang P, Urrea EM, Massari P, Mondragon-Ramirez G, Reyes-Acevedo R, Rice K, Rostaing L, Steinberg S, Xing J, Agarwal M, Harler MB, Charpentier B (2012). Three-year outcomes from BENEFIT, a randomized, active-controlled, parallel-group study in adult kidney transplant recipients. Am J Transplant.

